# Comprehensive Transcriptional Changes in the Liver of Kanglang White Minnow (*Anabarilius grahami*) in Response to the Infection of Parasite *Ichthyophthirius multifiliis*

**DOI:** 10.3390/ani10040681

**Published:** 2020-04-14

**Authors:** Ying Qiu, Yanhui Yin, Zhiqiang Ruan, Yu Gao, Chao Bian, Jieming Chen, Xiaoai Wang, Xiaofu Pan, Junxing Yang, Qiong Shi, Wansheng Jiang

**Affiliations:** 1 BGI Education Center, University of Chinese Academy of Sciences, Shenzhen 518083, China; qiuying@genomcis.cn (Y.Q.); ruanzhiqiang@genomics.cn (Z.R.); chenjieming@genomics.cn (J.C.); 2 Shenzhen Key Lab of Marine Genomics, Guangdong Provincial Key Lab of Molecular Breeding in Marine Economic Animals, BGI Academy of Marine Sciences, BGI Marine, BGI, Shenzhen 518083, China; bianchao@genomics.cn; 3 Yunnan Key Laboratory of Plateau Fish Breeding, Kunming Institute of Zoology, Chinese Academy of Sciences, Kunming 650223, China; yinyanhui@mail.kiz.ac.cn (Y.Y.); xueaiw@126.com (X.W.); xiaofupan@163.com (X.P.); 4 State Key Laboratory of Genetic Resources and Evolution, Kunming Institute of Zoology, The Innovative Academy of Seed Design, Chinese Academy of Sciences, Kunming 650223, China; 5 College of Animal Science and Technology, Yunnan Agricultural University, Kunming 650201, China; gaoyu@ynau.edu.cn; 6 Hunan Engineering Laboratory for Chinese Giant Salamander’s Resource Protection and Comprehensive Utilization, and Key Laboratory of Hunan Forest and Chemical Industry Engineering, Jishou University, Zhangjiajie 427000, China

**Keywords:** *Anabarilius grahami*, *Ichthyophthirius multifiliis*, transcriptome sequencing, immune response, antimicrobial peptide (AMP)

## Abstract

**Simple Summary:**

Kanglang white minnow (KWM, *Anabarilius grahami*), is a typical “3E” (Endangered, Endemic and Economic) fish species in Yunnan-Guizhou Plateau. As one of the traditional “Four Famous Fishes” in Yunnan province, it has become the major local aquaculture species with increasing demand after the success of artificial breeding. However, this economically important fish is highly susceptible to the infection of a parasite ciliate, *Ichthyophthirius multifiliis* (Ich), during the practical procedure of artificial breeding. To examine the host immune responses to Ich, we divided the experimental fishes into three groups (including control, early-infected stage, and late-infected stage) for transcriptome sequencing to analyze the differentially expressed genes (DEGs) and immune response mechanisms.

**Abstract:**

The notorious parasite *Ichthyophthirius multifiliis* (Ich) has been recorded worldwide in fish species and causes white spot disease, posing major threats and resulting in severe losses to international fish production. Extensively effective strategies for treating Ich are not available yet, and genetic mechanisms of hosts in response to the parasite are still largely unknown. In this study, we selected Kanglang white minnow (KWM, *Anabarilius grahami*) to examine its liver transcriptional changes after Ich infection, as white spot disease is one bottleneck problem in exploring this economically important species. We divided the experimental fishes into three groups (control, early-infected, and late-infected) to examine differentially expressed genes (DEGs). A total of 831 DEGs were identified and classified into 128 significantly enriched GO (Gene Ontology) terms and 71 significantly enriched KEGG (Kyoto Encyclopedia of Genes and Genomes) pathways. Most of these terms or pathways were functionally enriched in immunity, inflammatory response, and apoptosis, such as nucleotide-binding oligomerization domain-like (NOD-like) receptor signaling, tumor necrosis factor (TNF) signaling, interleukin-17 (IL-17) signaling, and apoptosis pathways. We also identified 178 putative antimicrobial peptides (AMPs) and AMP precursors based on our previously reported genome assembly of KWM, and revealed that the expressional patterns varied according to different types. In summary, our work reported the first comprehensive transcriptional changes in KWM in response to the exogenous infection of Ich, which would lay a solid foundation for in-depth studies on disease defense or resistant strains selection in this valuable fish.

## 1. Introduction

Kanglang white minnow (KWM), *Anabarilius grahami*, is a typical fish species with “3E” (Endangered, Endemic, and Economic) status and priorities in the Yunnan-Guizhou Plateau of China [1]. It has been known as one of the “Four Famous Fishes” in Yunnan, with a restricted distribution in Fuxian Lake, the second deepest lake in China [2]. In spite of its small size, KWM was once the major economically important fish from the Fuxian Lake, accounting for up to 70%–80% of the natural fishery production before the 1990s [3]. However, since then the wild population of KMW has decreased sharply due to the introduction of an exotic icefish (*Neosalanx taihuensis*) [4], along with overfishing, destruction of spawning sites and the low fecundity of KMW itself [1]. These anthropogenic causes shifted the KMW from being an abundant economic species to an endangered fish, with evaluations to vulnerable (VU) in the China Species Red List in 2004 [5] and 2015 [6]. To preserve the KWM from extinction, artificial breeding was achieved by us in 2003 [7], which has ensured the gradual reintroduction of its wild populations, and also created a new chance for aquaculture utilization of this valuable species [1]. However, farm-raised KMW are susceptible to exogenous infections of *Ichthyophthirius multifiliis* (Ich), which has caused heavy damages every year and become one of the major bottleneck problems in the recent exploration of this plateau fish species.

Ichthyophthiriasis caused by Ich has been recorded extensively in fish species worldwide, and is commonly referred to white spot disease. Ich has acted as the most prevalent and severe parasite of freshwater fishes [8,9], and generated great economic losses in both food and aquarium fish production [10]. Ich usually invades the epithelial layer of the gill and skin, and triggers both innate and adaptive immune responses in its hosts, locally and systemically [11]. Its infection usually leads to high morbidity and mortality rates in farming fishes [12]. Although the Ich only has a simple life cycle, including an infective stage of theronts, a parasitic stage of trophonts, and a reproductive stage of tomites [13], extensively effective strategies for treatment are far from resolved. At present, most treatments of Ich infection are from chemicals, however, it is difficult to eliminate the consequent food safety and environmental pollution issues. A growing number of studies have focused on anti-parasitic responses in genes and pathways while host defenses against Ich infection, and they are expected to provide novel insights into the treatment of Ich infection in practical aquaculture. Immune responses to Ich infection have been examined initially in some fish species, such as zebrafish [14], channel catfish [15,16], Nile tilapia [17] and carps [18,19,20,21]. However, most previous studies have only examined the transcriptional changes of some specific and well-known immune related genes or pathways under Ich infection, such as toll-like receptor genes (TLRs), major histocompatibility complex (MHC) molecules, inflammatory cytokines and immunoglobulins [14,21,22].

Antimicrobial peptides (AMPs) are a group of amphiphilic and cationic short peptides, which exist widely in various animals with comprehensive biological activities and serve as critical components in immune systems against infectious pathogens [23]. Since the importance of AMPs has been gradually recognized and studied, the public online Antimicrobial Peptide Database (APD, 2016 version, http://aps.unmc.edu/AP/main.php) has contained 3138 AMPs from six kingdoms. AMPs usually have specific gene structures or come from the hydrolysis of some immune-related proteins, like histone, hemoglobin, thrombin, etc. [24]. In recent years, many AMPs have been identified by us through genome and transcriptome screening in fishes, such as amphibious mudskippers [25], line seahorse [24] and giant grouper [26]. Interestingly, our previous antibacterial experiments using AMPs/AMP precursors from mudskippers have revealed their efficiencies in inhibiting the growth of *Micrococcus luteus* [25], which suggested that AMPs might play some roles in KWM against infectious pathogens such as Ich.

High-throughput transcriptome sequencing is a large-scale, efficient, and economical method for functional gene identification, and has been proved to be valuable in finding of candidate genes from fish disease studies [27,28,29]. However, transcriptome data of KWM are still unavailable, especially immune mechanisms to the current bottleneck problem of Ich infection remain unknown. For in-depth understanding of the molecular responses of immune systems in KMW, we conducted three pairwise comparisons between control, early and late Ich infected stages, and analyzed immune-related genes and signaling pathways subsequently. In addition, we also identified AMPs/AMP precursors from our previously reported genome assembly of KWM [1], and analyzed their specific transcriptional changes among the three comparative fish groups by a combination of the genome and transcriptome data in this study.

## 2. Materials and Methods

### 2.1. Fish and Parasite Resources

Twenty-eight parasite-free, similar-sized (around 160 mm in length), and healthy KWM fishes were selected from our artificially cultivated stocks in the Endangered Fish Conservation Center (EFCC) of the Kunming Institute of Zoology (KIZ), Chinese Academy of Sciences, Kunming, Yunnan Province, China. Parasite ciliate Ich was obtained from some highly infected goldfishes, which were picked from a local ornamental fish market. The KWM and goldfishes were transferred and kept in two separated experimental tanks in our laboratory of KIZ for subsequent use. All animal experiments were approved by the internal review board of KIZ (approval ID: 2015-SMKX026).

### 2.2. Experimental Design

After being temporarily acclimated for two days, 28 KWM fishes were assigned randomly into two experimental tanks to decrease the rearing density (each held 14 individuals). Tissue samples were collected from three individuals of each tank as the control group before the Ich challenge. Subsequently, one goldfish with heavy infection by Ich (white spots with dense cover on the skins and fins, and the parasite was verified to be trophonts of Ich by microscope examination) was put into each tank. Soon after, the symptom of white spots on fins and skins appeared in some KWM at the 4th day post-infection (dpi). Therefore, we collected samples from these KWM fishes that showed less than 10 white spots on the skins and fins (verified to be Ich by microscopic examination) but with normal swimming behaviors. We defined them as the group of early-infected stage. Successively, a few fishes died but without any symptoms of white spots, or died sporadically during our observation without collecting samples from dead fishes. On the 17th dpi, some fishes were observed with increasing white spots on trunks and fins. We applied full time observation to these fishes and collected samples at the time when they lost balance in swimming but were still alive. We defined the fishes at this period as the late-infected stage. In total, 6, 3, and 3 samples were collected (met the conditions we defined) in the groups of control, early-infected stage, and late-infected stage, respectively (Figure S1). It is worth noting that the control group in our design represented the time zero samples before infection, and we did not trace any other control samples at the same time when we collected samples in the early- and late-infected stages. Liver, head kidney, intestine, gonad, gill and muscular tissues were collected from each fish, and then transferred to liquid nitrogen for storage immediately. The liver samples of three individuals (designed as biological replicates) in each of the three groups were performed with high-throughput transcriptome sequencing, and the rest of the samples were reserved for standby using.

### 2.3. RNA Extraction, Library Construction and Transcriptome Sequencing

A total of nine fishes were used for transcriptome sequencing, with an average standard length of 153.2 ± 13.0 mm and body weight of 33.4 ± 10.9 g. Total RNA was extracted individually using TRIzol Reagent (Invitrogen, CA, USA), and then treated with RNase-free DNase I (Thermo Scientific, CA, USA). The RNA Integrity Number (RIN) values of the nine RNA samples ranged from 8.7 to 9.3 and were evaluated with high qualities. Nine sequencing libraries from the liver samples were sequenced on an Illumina HiSeq X Ten platform with 2 × 150 bp paired-end (PE) reads by BGI (BGI-Shenzhen, Shenzhen, China).

### 2.4. Read Alignment and Gene Expression Analysis

Raw data were filtered with removal of adaptor sequences, contaminations and low-quality reads (quality score ≤ 20) using SOAPnuke (v1.5.6, filter—nRate 0.05—lowQual 20) [30]. We employed our previously published genome sequences of KWM [1] as the reference. Cleaned reads were aligned to the reference using HISAT2 (v2.0.4) [31]. Gene transcriptional levels were estimated using in-house Perl scripts, and RPKM (reads per kilobase of exon per million fragments mapped) values were calculated to represent the relative mRNA levels for each transcript.

### 2.5. Gene Expression Analysis and Enrichment Analysis

We conducted three pairwise comparisons between control, early- and late-infected stages. Gene transcriptions were determined using three different methods including DESeq2 [32], EBSeq [33] and NOISeq [34], while the differentially expressed genes (DEGs) were defined as these genes with log2 (RPKM) values > 2 and adjusted *p*-value < 0.05 [35]. The GO (Gene Ontology) enrichment analysis of DEGs was performed by the EnrichPipeline [36,37]. The enrichment analysis of KEGG (Kyoto Encyclopedia of Genes and Genomes) pathways was implemented using KOBAS (Release 81.0) [38].

### 2.6. Validation of Gene Transcription Using Quantitative Real-Time Polymerase Chain Reaction (qRT-PCR)

We selected several representative DEGs from each pairwise comparison to carry out a further quantitative real-time polymerase chain reaction (qRT-PCR) amplification to validate the transcriptome results. The qRT-PCRs were performed on an ABI StepOnePlus Real-Time PCR System (Applied Biosystems, CA, USA) with SYBR Green as the fluorescent dye (Sangon Biotech, Shanghai, China) according to the manufacturers’ instructions. Primer sequences (Table S1) were designed using the Primer Premier 5 software (Primer Biosoft, CA, USA). PCR reactions were conducted in a total volume of 20 μL and the conditions were set as follows: an initial denaturation at 95 °C for 3 min, followed by 40 cycles of denaturation at 95 °C for 10 s, annealing at 60 °C for 30 s, and extension at 72 °C for 10 s. All reactions were performed in triplicate to provide technical repeats, and the relative expression levels were normalized with β-actin for each sample by using the 2^ΔΔCT^ method [39].

### 2.7. Identification of Antimicrobial Peptides (AMPs)

Firstly, we downloaded 2624 AMP amino acid sequences from the online Antimicrobial Peptide Database (APD, 2016 version, http://aps.unmc.edu/AP/main.php) and applied homology searches to predict AMPs/AMP precursors. Secondly, we employed Protein-protein Basic Local Alignment Search (BLASTP) at an E-value ≤ 1 × 10^−5^ to map these predicted AMP sequences onto our previously annotated gene set of KWM [1], and filtered those hits with query alignment ratio less than 0.5. Thirdly, we used KOBAS (Release 81.0) [38] to implement the KEGG pathways enrichment analysis of the predicted AMPs/AMP precursors. Finally, we extracted RPKM values for the identified AMPs/AMP precursors from the gene expression analysis and compared their transcription patterns among the three experimental groups of KWM in response to the infection of Ich.

## 3. Results

### 3.1. Identification and Analysis of Differentially Expressed Genes (DEGs)

**Figure 1:**
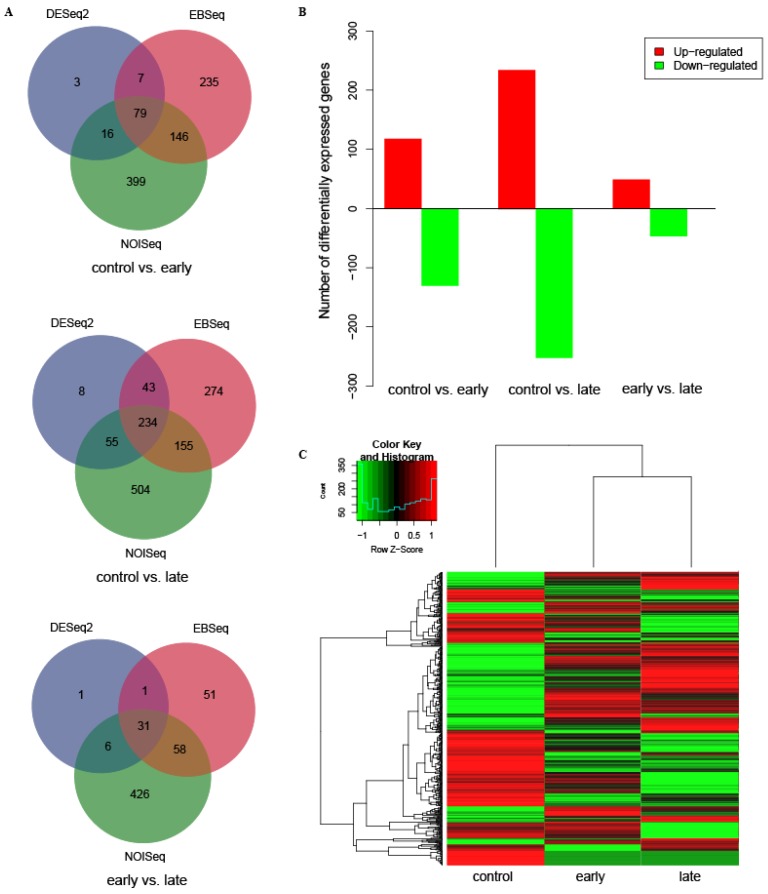
Summary of DEGs identified by three analysis methods within three comparative groups. (**A**) The number of DEGs identified by three analysis methods in the three comparative groups. (**B**) The number of up-regulated and down-regulated genes in the three comparative groups. (**C**) A hierarchical clustering of DEGs identified in the three comparative groups. Scale of the color bar represents fold changes of transcription levels.

To reveal the potential immune mechanisms of KWM to Ich infection, we carried out transcriptome sequencing on liver samples from three controls, three early-infected and three late-infected fishes. In total, we obtained 718 million raw reads, which were deposited in National Center for Biotechnology Information (NCBI) Sequence Read Archive (SRA) under accession number SRP237833. After quality control, nine libraries of clean reads (694 million) were mapped back to the reference genome assembly [1] and then were used to calculate the transcription levels of genes. The DEGs from pairwise comparisons of controls, early- and late-infected fishes, have showed varied numbers. In generally, they decreased in the following order: NOISeq > EBSeq > DESeq2 (Figure 1A). For this reason, we only chose those DEGs with overlaps in any two methods to represent the final DEGs for subsequent analyses.

**Figure 2:**
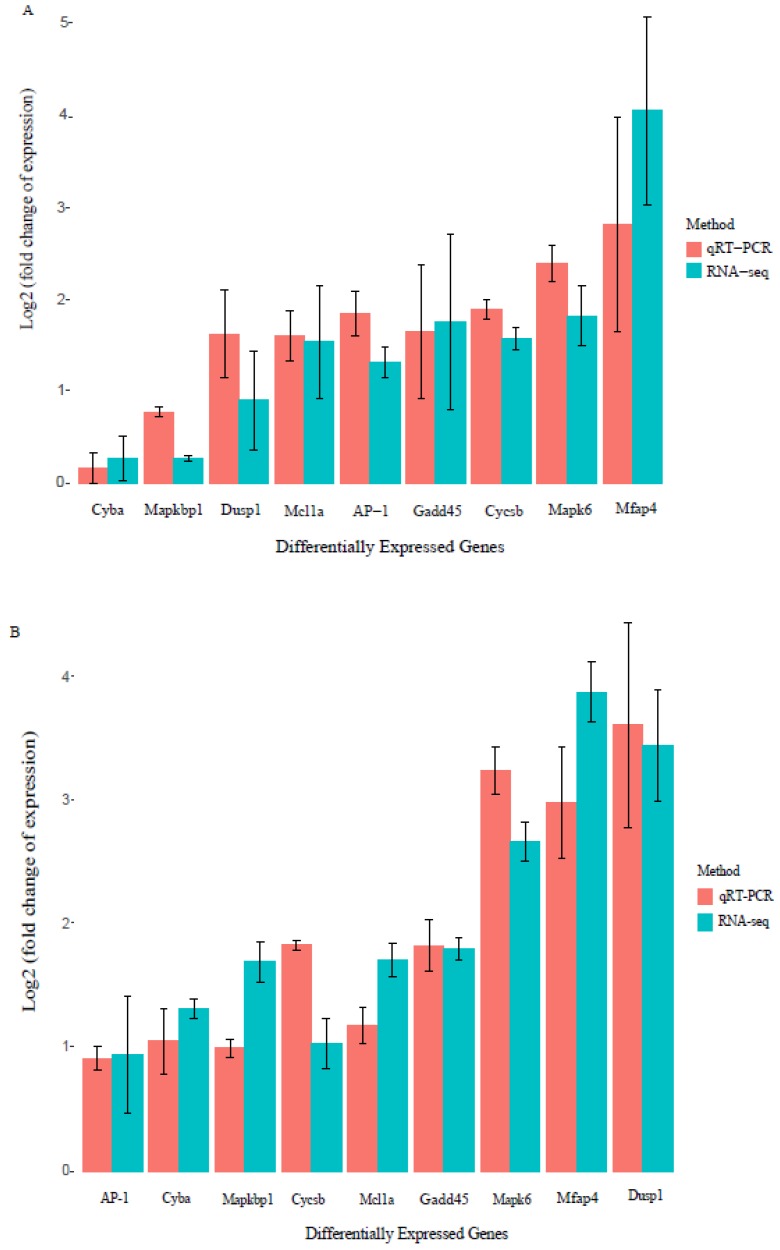
Validation of transcriptome results by quantitative real-time polymerase chain reaction (qRT-PCR). Bars showed the log2-fold change values of nine randomly selected DEGs in two pairwise comparisons: control vs. early-infected stage (**A**) and control vs. late-infected stage (**B**). Red columns represent the transcriptional fold changes measured by qRT-PCR, and green columns indicate the RPKM (reads per kilobase of exon per million fragments mapped) values measured by the transcriptome sequencing. Error bar of each column represents the standard error of the mean from three independent experimental replicates.

Overall, 831 DEGs were finally identified from KWM in response to the infection of Ich. Among them, 402 were up-regulated (*p* < 0.05), comprising 118 (control vs. early-infected), 235 (control vs. late-infected) and 49 (early vs. late-infected) respectively in the three pairwise comparisons. The number of down-regulated genes (*p* < 0.05) was 429, including 130 (control vs. early-infected), 252 (control vs. late-infected) and 47 (early vs. late-infected) respectively in the three pairwise comparisons (Table 1). Relatively, the number of DEGs between control vs. late-infected group was the most abundant, while these between early vs. late-infected group was the least (Figure 1B). A hierarchy clustering analysis on the transcription levels of DEGs among the three fish groups showed that the transcription patterns of the early- and late-infected were more similar (clustered together, Figure 1C). Additionally, we randomly selected nine DEGs to perform a qRT-PCR analysis. The transcription levels from the qRT-PCR showed high consistency with the transcriptome sequencing patterns (see more details in Figure 2), with all of the correlation coefficients being greater than 0.85 (*p* < 0.01, Figure S2), thus suggesting the reliability of our high-throughput sequencing data generated in this study.

### 3.2. Functional Analyses of DEGs Based on GO (Gene Ontology) and KEGG (Kyoto Encyclopedia of Genes and Genomes) Enrichments

**Figure 3:**
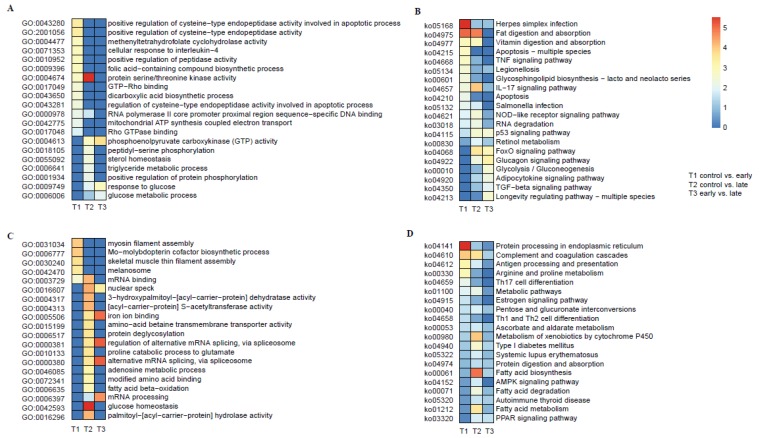
Functional analyses of the top 20 GO and KEGG enrichments based on DEGs from three comparative groups. (**A**) Enriched GO terms for the up-regulated DEGs after the *Ichthyophthirius multifiliis* (Ich) infections. (**B**) Enriched KEGG pathways for the up-regulated DEGs after the Ich infections. (**C**) Enriched GO terms for the down-regulated DEGs after the Ich infections. (**D**) Enriched KEGG pathways for the down-regulated DEGs after the Ich infections.

These DEGs triggered by Ich infection are supposed to be the key genes for immune response. To reveal the detailed mechanisms, we performed GO enrichment analysis. In total, 831 DEGs were classified into 1582 GO terms, including 128 significantly enriched ones (*p*-value < 0.05, Table 1). The up- and down-regulated DEGs in the three pairwise comparisons (control vs. early-infected, control vs. late-infected, early vs. late-infected groups) were enriched into 87 and 41 GO terms, respectively (Figure 3A,C, Tables S3 and S4). When considering the biological functions, we corelated the GO terms of up-regulated DEGs with immunity, apoptotic and metabolic, such as ‘cellular response to interleukin-4′ (GO:0071353), ‘regulation of cysteine-type endopeptidase activity involved in apoptotic process’ (GO:0006915), ‘organic cyclic compound metabolic process’ (GO:1901360) and ‘macromolecule metabolic process’ (GO:0043170). On the other hand, the GO terms of down-regulated DEGs were enriched in metabolic functions. These DEGs enriched in apoptotic and metabolic functions suggested that the parasite infection had strong impacts on the cell apoptotic and energy demand of the host (Table S2).

Additionally, to identify the biological pathways that were activated in KWM in response to Ich infection, we classified DEGs of the three pairwise comparisons into 284 KEGG pathways, including 71 significantly enriched ones (*p*-value < 0.05, Table 1). We mapped the up- and down-regulated DEGs into KEGG pathways separately (Figure 3B,D, Tables S5 and S6). Interestingly, the significantly enriched pathways of the up-regulated DEGs focused on immunity, inflammatory response and apoptosis, such as nucleotide-binding oligomerization domain-like (NOD-like) receptor (NLR) signaling pathway, tumor necrosis factor (TNF) signaling pathway, interleukin-17 (IL-17) signaling pathway, and apoptosis. Some KEGG pathways enriched from the down-regulated DEGs, however, also related to immune response, such as antigen processing, Complement and coagulation, Th17, Th1 and Th2 cell differentiation as well as the Peroxisome proliferator-activated receptor (PPAR) signaling pathway.

### 3.3. Transcriptional Patterns of the DEGs with Immune Related Functions

The NLR signaling pathway is responsible for detection of various pathogens and generation of innate immune responses. We found that 12 genes in the NLR signaling pathway were significantly up-regulated. For example, the transcription levels of transcription factor AP-1 and pyrin domain-containing (PYD-containing) protein (*NLRP3* and *NLRP12*) were up-regulated significantly in the early- and late-infected fishes. These genes were well-known to participate in many immunity and apoptosis related pathways, such as NLR signaling pathway, Mitogen-activated protein kinase (MAPK) signaling pathway, TNF signaling pathway and apoptosis. *MyD88*, potentially involved in the toll-like and apoptosis pathway, was also significantly up-regulated following the Ich infection.

DEGs of up-regulated in the pairwise comparisons were also enriched in TNF signaling pathway and interleukin-17 (*IL-17*) signaling pathway. For example, the transcription level of interleukin 17 receptor E (*IL-17RE*) was up-regulated in the early- and late-infected fishes. Additionally, we observed that the interleukin enhancer binding factor 3b (*Ilf-3b*), nuclear factor interleukin 3 regulated member 6 (*Nfil3-6*) and interferon regulatory factor 2 binding protein 2b (*Ifr2bp2b*) were also up-regulated in the early and late infected fishes.

Additionally, among the 831 DEGs, we found some immune-related genes with significantly high transcription levels although without enrichments in the KEGG pathways. Transcription levels of dual-specificity phosphatase 1 (*Dusp1*), microfibrillar-associated protein 4 (*Mfap4*) and G-protein-coupled receptor 75 (*Gpr75*) were up-regulated in the early- and late-infected fishes. The transcription level of mitogen-activated protein kinase binding protein (*Mapkbp1*), a key transcription factor in the NF-κB signaling pathway [40], was also identified to be up-regulated after Ich infection.

Simultaneously, we also identified some down-regulated DEGs with functions related to immune pathways, such as acyl-CoA synthetase long-chain family member 5 (*Acsl5*), interleukin 6 signal transducer (*Il6st*) and heat shock protein 90 beta member 1 (*Hsp90b1*).

### 3.4. Identification of AMPs and Their Transcriptional Analysis

**Figure 4:**
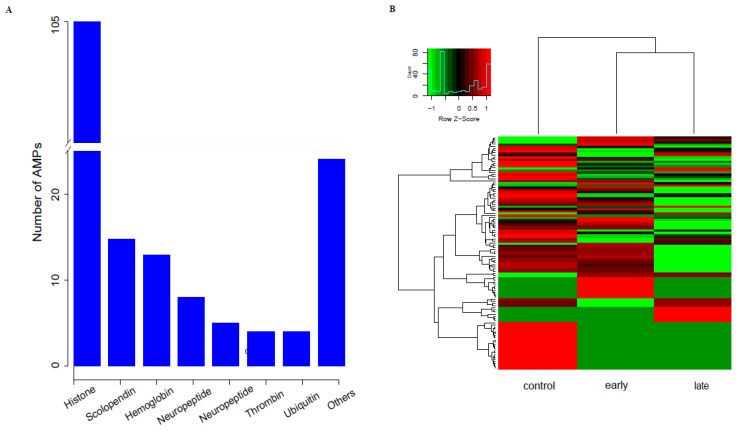
The summary of antimicrobial peptides (AMPs)/AMP precursors in Kanglang white minnow (KWM) in response to the Ich infections. (**A**) Confirmed peptides number hit against the gene set in KWM according to the different type of AMPs/AMP precursors (Others includes all other type that with only one or two confirmed peptides under that type). (**B**) Transcriptional variances of identified AMPs/AMP precursors in KWM in response to the Ich infections. Scale of the color bar represents fold changes of transcription levels in three groups.

To identify the putative AMP sequences, we employed BLASTP to search against our previously annotated gene set, using those AMP sequences downloaded from the public APD database [41] as the queries. In total, we obtained 178 putative AMP sequences in KWM, and they were classified into 24 groups, including 105 histones, 15 scolopendins, 13 hemoglobins, 8 neuropeptides, 5 chemokines, 4 thrombins, 4 ubiquitins and 17 other types (each with only one or two AMPs, Figure 4A, see more details in Tables S7 and S8). These AMP sequences were enriched into 96 KEGG pathways. Representative pathway clusters included “Immune diseases”, “Substance dependence”, “Cancers: Overview” and “Immune system” (Figure S3), which indicated that most of the putative AMPs/AMP precursors were potentially involved in immune response and disease defense. In addition, we noticed that 8, 2 and 2 putative AMP/AMP precursor genes were clustered into “Infectious diseases: Viral”, “Infectious diseases: Bacterial” and “Infectious diseases: Parasitic” terms, respectively, which were highly relevant to antimicrobial defense.

Corresponding transcription levels of these genes were extracted for calculating their RPKM values. Interestingly, relative to the control (non-infection), the transcription levels of some AMPs/AMP precursors were down-regulated at the early- and late-infected stages (see more details in Figure 4B). For instance, we analyzed the top 20 putative AMPs/AMP precursors based on the transcription levels (RPKM values) at different stages. The histone family, one major type of the AMPs/AMP precursors that we identified in KWM, was generally down-regulated at the early- and late-infected stages (Table S9). However, the RPKM values of the scolopendin, chemokine and thrombin family were relatively up-regulated at the early- and late-infected stages. In particular one type of AMP, the *Alpha-1-antiproteinase*, was up-regulated at the early-infected stage but down-regulated following the late infection, and its transcription level was several orders of magnitude greater than the other AMPs/AMP precursors. Additionally, the *Amylin*, which showed no transcription in control and early-infected fishes but transcribed at late-infected stage was also revealed (Table S9).

## 4. Discussion

A sound experiment design about fish disease should leave the fishes only under the pressure of challenging pathogens, however, this goal is difficult to achieve as other stresses would produce on the remaining fishes when some fishes died in the experimental tanks, and other conditions might alter such as the water microenvironment might also have undergone light modifications during the infection trial. We have to admit that we did not cover these issues, and the control group in our study only represented the samples at time zero before infection but not the simultaneous non-infected control samples that should be collected at the same time as early- or later-infected stages. Using time zero as the control seems like a simply compromise choice, but it has been widely accepted for pathogen challenging studies in various fishes [19,20,29,42].

For comparative transcriptome analyses, identification of DEGs was the most fundamental task for revealing molecular mechanisms under the specifically phenotypic variations or experimental designs. Various expression analysis methods have been developed with different algorithms, such as the DESeq2 that was based on negative binomial distributions [32], the EBSeq representing a Bayesian approach with a negative binomial model [33], and the NOISeq that was adapted from non-parametric methods [34]. Usually, researchers only choose one method to run through all the necessary analyses. To improve the reliability of identified DEGs, we employed a strategy to minimize the method biases by extraction of those DEGs that with overlaps between any two methods. In this strategy, we identified a total of 831 genes from the three pairwise comparisons of controls, early- and late-infected fishes, which were expected to be the most representative DGEs of KWM in response to the infection of Ich.

The number of DEGs between the groups of control vs. late-infected was largely greater than the groups of early- vs. late-infected (Figure 1B). The hierarchy clustering analysis also showed that the transcription patterns of early- and late-infected fishes were more alike (Figure 1C). It suggested that most relevant genes had been triggered even in a short period in response to the infection, implying that KWM is highly sensitive to Ich. Meanwhile, as the infection is going on deeply, more DEGs were activated to defend pathogens, and unsurprisingly, most of these up-regulated DEGs were enriched into GO terms or pathways with immune-related functions. For instance, many of the activated DEGs were enriched in the NLR signaling pathway, suggesting their vital functions for KWM in defensing against the Ich. As we know, innate immune response is initiated through recognition of exogenous pathogens by the pattern recognition receptors (*PRRs*) [43], NOD-like receptors (*NLRs*) and Toll-like receptors (*TLRs*) are two major components of the PRRs that provide immediate responses against pathogenic infection or tissue injury [44]. Similar immune response was also reported in Przewalskii’s naked carp (*Gymnocypris przewaliskii*) while in response to Ich infection [45]. Although TLRs were not detected to be significantly enriched in the present study, 12 genes in the NLR signaling pathway were significantly up-regulated. For KWM, the NLRs would possibly cooperate with TLRs to defense the infections of Ich.

The TNF signaling pathway and *IL-17* signaling pathway were detected to be significantly up-regulated after the infection of Ich. Interleukins (*ILs*) and interferons (*IFNs*), belonging to cytokines, are produced by granulocytes, macrophages, lymphocytes or epithelial cells [46]. Cytokines are usually secreted by activated immune-related cells after infected with various pathogens [47]. The *IL-17* family members, especially, played an important role in the maintenance of mucosal homeostasis and mucosal immunity [48]. In this study, certain cytokine genes, such as *IL-17RE, Ilf-3b, Nfil3-6* and *Fr2bp2b*, were up-regulated in the early- and late-infected fishes. It had been reported that the expression of inflammatory cytokine genes like *TNFα, IL-1β, IL-6* and *IL-8* were up-regulated by the infection of Ich in rainbow trout larvae [22]. In common carp, Gonzalez et al. [21] also reported significant transcriptional increase of *IL-1β* and *TNF-α* genes after the infection of Ich. In a word, both our and previous studies indicated that the cytokine genes were widely involved in the innate immune responses.

Cell apoptosis related genes was usually activated after disease infection [29]. In this study, we found the up-regulated DEGs in apoptosis (ko04210) has the highest transcription levels at the early-infected stage, and then slightly decreased in the late-infected fishes. Apoptosis is a genetically programmed process for elimination of damaged or redundant cells by activation of caspases. When at the early-infected stage, the infected fishes presented severe immune responses and the transcriptions of apoptosis-related genes (such as *Gadd45* and *c-jun*) increased more than three-fold compared with the control fishes. With the resistance to parasite infection and self-protection, the transcriptions of apoptosis-related genes decreased slightly along time.

For specific genes, the significantly high transcription levels of some well-known immune-related genes (such as *Dusp1, Mfap4, Gpr75* and *Mapkbp1*) in KWM were emphasized in this study. Mapkbp1 is a key transcription factor in the NF-κB signaling pathway, and NF-κB is a family of inducible transcription factors that regulate innate and adaptive immune functions and inflammatory responses [49,50]. On one hand, it suggests that these genes may play vital roles in innate immune responses. On the other hand, it shows high consistency with the previous idea that fishes would develop strong innate immune responses when infected with Ich [51].

Nevertheless, we also paid much attention to some of the down-regulated genes with immune-related functions but without enough attentions before, such as *Acsl5, Il6st and Hsp90b1*. *Acsl5* has been reported not only involved in fatty acid uptake, mitochondrial metabolism but also associated with decreased Wnt activity and increased expression in CD8 and CD4 T-cells [52]. *Il6st* plays a pleiotropic effect on immune response, tissue injuries, inflammation and hematopoiesis [53]. An immune-stimulation study of Avian Macrophages suggested the involvement of *Hsp90* and kinase (MAPK/extracellular signal-regulated kinases (ERK) and Phosphoinositide 3-kinase(PI3K)/ Protein Kinase B (AKT)) pathways in CpG mediated immune-stimulation [54]. Since some enriched pathways from down-regulated DEGs were also involved in immune response (Figure 3D), these results suggested that Ich would possibly dismiss the immune response of KWM somehow. For reverse thinking, these down-regulated immune-related genes would be treated as the regulative targeted genes in the future preventive treatment or resistant strains selection.

In considering that AMPs served as critical components in immune systems and in order to study the expressional patterns of AMPs in KWM in response to the infection of Ich, we identified AMPs of KWM by analyzing our genome data [1] firstly, and then compared the specific transcriptional changes among the experimental groups in this study. As we know, most AMPs can be classified into two main types. One type has been characterized by their functions illustrated from the gene names (such as cathelicidin and piscidin), while the other one is usually derived by the proteolysis of immune related genes (such as cytokine, thrombin, histone, chemokine and lectin) [25]. Histones are mediators of thrombosis and also serve as microbicidal, connecting the immune system to the coagulation system [55,56]. In KWM, 115 peptides of histones were identified and served as the major sources of AMPs. However, these histones were detected to be generally down-regulated at the early- and late-infected stages. In contrast, the immune-related AMPs such as the scolopendin, chemokine and thrombin family were up-regulated after Ich infection in KWM. Scolopendin is known to be active in innate immunity with regulatory functions, and our previous study showed that the scolopendin-derived putative AMPs were one of the three largest parts in giant grouper (*Epinephelus lanceolatus*) [26]. The up-regulation of scolopendin, as well as chemokine and thrombin after Ich infection suggested these that AMPs were widely involved in the innate immune responses.

In addition, *Alpha-1-antiproteinase* (also known as alpha-1-antitrypsin), a major inhibitor present in human body that has been related to anti-inflammatory immune response [57], showed a relatively high transcription with RPKM values ranging from 39,053.34 to 60,744.41. It indicated a very intensive anti-inflammatory immune response of KWM in defensing with the Ich infection. Our previous antimicrobial assays of the amyloid genes in mudskipper (*Boleophthalmus pectinirostris*) revealed that amylin has a strong antibacterial activity against the infection of Gram-positive bacterium *M. luteus* [25]. In this study, amylin (annotated to *amylin-BP*) showed no transcription in control and early-infected fishes but expressed at late-infected stage (Table S9), indicating that the KWM would suffer co-infection with bacteria at late time during Ich infection. In short, the transcription patterns of AMPs/AMP precursors were variable according to different types, thus indicating the mechanisms of AMPs against Ich infection were complicated. Our present study identified, for the first time, the AMPs/AMP precursors and their corresponding transcriptions in KWM in response to Ich infection, which provided valuable AMPs resources for further exploration and application in this Yunnan-Guizhou Plateau “3E” fish.

## 5. Conclusions

In this study, we identified 831 genes that were significantly differentially expressed in KWM in response to the infection of Ich, including 402 activated and 429 suppressed genes. We also revealed that the up-regulated genes mainly participated in the pathways related to immunity, inflammatory response and apoptosis, such as NLR signaling, TNF signaling, IL-17 signaling and apoptosis pathways. The suppressed genes were mainly enriched in metabolic functions, but also some enriched in immune-related pathways that might indicate an Ich dismissed immune response. Meanwhile, we identified 178 putative AMPs/AMP precursors in KWM and found that their transcriptional levels were variable according to different types, suggesting the complex mechanisms of AMPs/AMP precursors against Ich infection. In conclusion, this work described the first transcriptional profiles of genes in three different groups (control, early- and late-infected), and proposed the potential molecular mechanisms of immune responses of KWM to exogenous infection of Ich.

## Figures and Tables

**Table 1:** Number of differentially expressed genes (DEGs), Gene Ontology (GO) terms and Kyoto Encyclopedia of Genes and Genomes (KEGG) pathways (*p*-value < 0.05) from the pairwise comparisons.

Pairwise Comparison	DEGs			GO Terms				KEGG Pathway
	Up-Regulated	Down-Regulated	Total	Biological Process	Cellular Component	Molecular Function	**Total**	
control vs. early-infected	118	130	248	20	1	11	32	30
control vs. late-infected	235	252	487	54	1	23	78	28
early vs. late-infected	49	47	96	11	1	19	31	18
Total	402	429	831	85	3	53	128 *	71 *

DEGs up-regulated or down-regulated represent that the latter group was up-regulated or down-regulated relative to the former group in each pairwise comparison. * The total number was less than the sum of each line because we removed the overlaps among the three pairwise comparisons.

## Supplementary Materials

The following are available online at https://www.mdpi.com/2076-2615/10/4/681/s1: Table S1: Primers for qRT-PCR verification. Table S2: Apoptotic and metabolic related GO terms with number of DEGs ≥ 2. Table S3: Enriched GO terms for the up-regulated DEGs after the Ich infections. Table S4: Enriched GO terms for the down-regulated DEGs after the Ich infections. Table S5: Enriched KEGG pathways for the up-regulated DEGs after the Ich infections. Table S6: Enriched KEGG pathways for the down-regulated DEGs after the Ich infections. Table S7: Summary of categorization about identified putative AMPs/AMP precursors. Table S8: Summary of the 178 identified putative AMPs/AMP precursors. Table S9: Top 20 putative AMPs/AMP precursors based on the transcription levels (RPKM values) at three different stages. Figure S1: The tracing number of survived, dead (including samples), and dead fish verified by infection of Ich. Figure S2: Correlation of gene transcription obtained from qRT-PCR and RNA-seq in control vs. early-infected fishes (A) and control vs. late-infected fishes (B). Figure S3: KEGG annotation of the putative AMP/AMP precursor genes in KWM.
