# Supplementary material for: Comprehensive Transcriptional Changes in the Liver of Kanglang White Minnow (Anabarilius grahami) in Response to the Infection of Parasite Ichthyophthirius multifiliis
Source: Animals (Basel). 2020 Apr 14;10(4):681. doi: 10.3390/ani10040681 (PMC7222788; doi:10.3390/ani10040681)
Supplement: Supplementary file 1 [file animals-10-00681-s001.zip › Supplementary Materials.docx]

**Supplementary Materials**


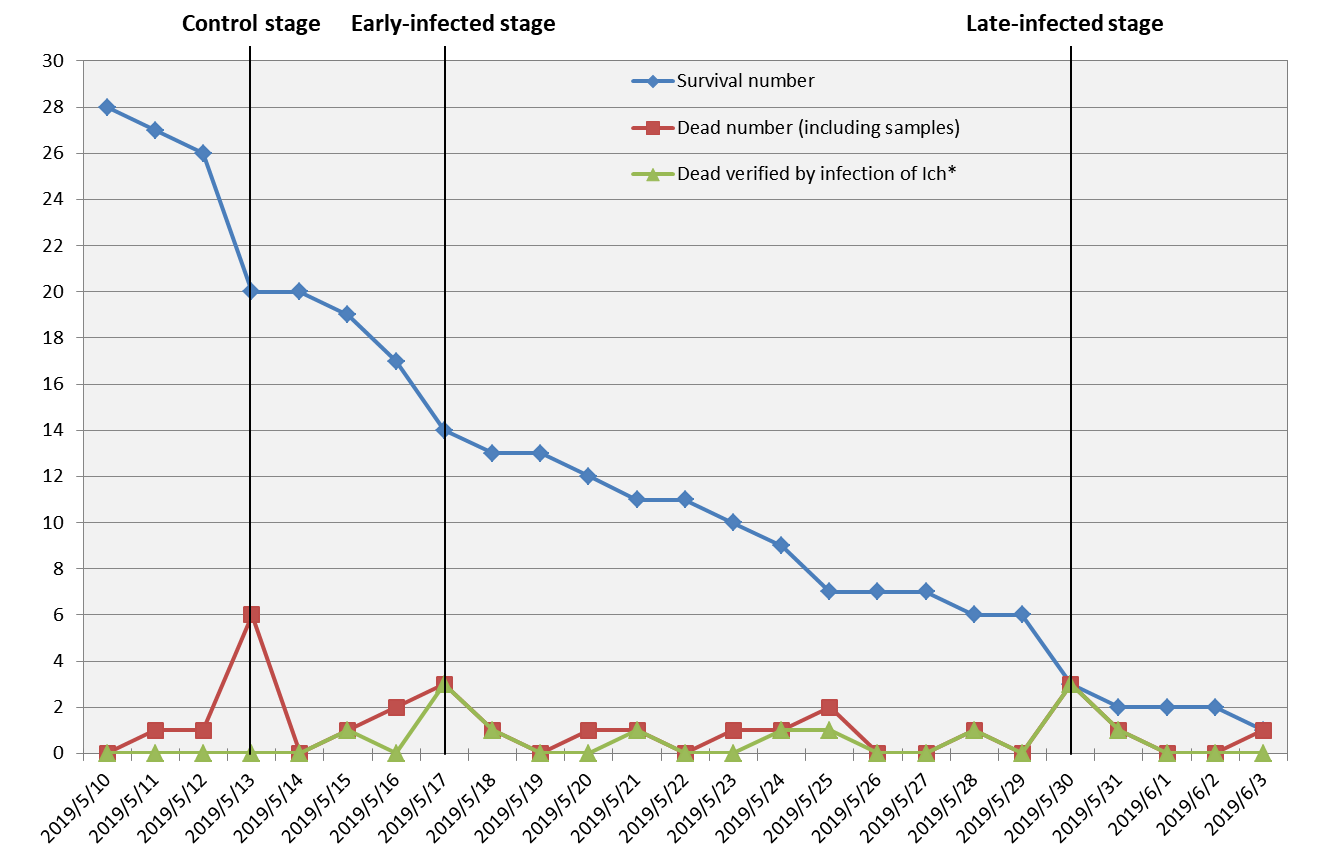


**Figure S1** The tracing number of survived, dead (including samples), and dead fish verified by infection of Ich (*, with white spot symptoms and examined to be Ich by microscope).


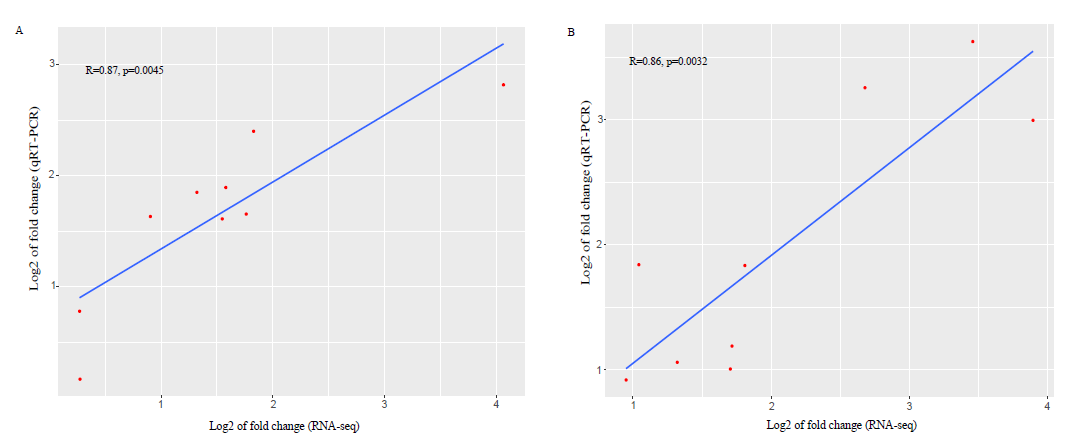


**Figure S2** Correlation of gene transcription results obtained from qRT-PCR and RNA-seq in control vs. early-infected fishes (A) and control vs. late-infected fishes (B).


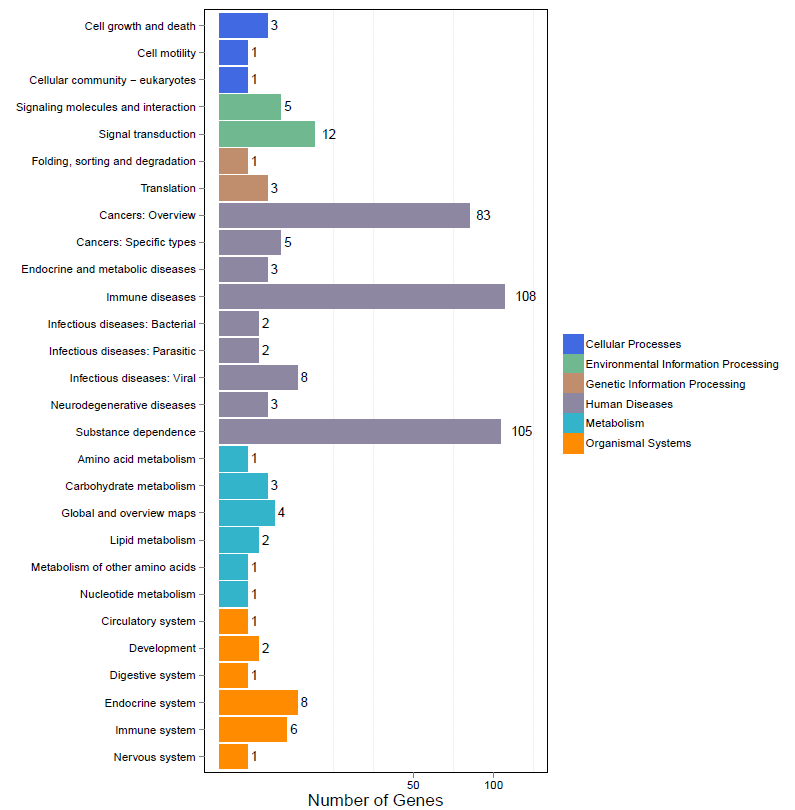


**Figure S3** KEGG annotation of the putative AMP/AMP precursor genes in KWM (A total of 178 predicted genes were clustered into 96 KEGG pathways).

**Table S1** Primers for qRT-PCR detection

| Primers | Sequences (5’-3’) |
| --- | --- |
| β-actin-F | CCAAAGCCAACAGGGAAAAG |
| β-actin-R | ACCAGAGGCATACAGGGACAG |
| Gadd45-F | GTCGGCGTTTATGAGTCTGC |
| Gadd45-R | AATGAGCGTGAAGTGAATCTGC |
| Dusp1-F | TCAGCTCGTCTCATATTTCGG |
| Dusp1-R | TTCTTCACTTCTCCCATCTCCA |
| Mapk6-F | ACTGGTCTTTTCCGCTGTCG |
| Mapk6-R | TCTGCGTATGATTTTGATTTCG |
| Cyba-F | TGGACTCATTTATGCGACTGG |
| Cyba-R | AACGCTCTGACGTAATAGTTTCTG |
| Mapkbp1-F | ATTTATTTCCAGCACGAAGGTC |
| Mapkbp1-R | TGGTGGTGGGGCTCATGTAG |
| Mcl1a-F | AACGAACGGGCTGAAAGGAC |
| Mcl1a-R | GTGCGATAGAAATCCAACAAAAG |
| Mfap4-F | AACTGTGCCAAACTGTATCTCGG |
| Mfap4-R | GGTTGACCAAACAGCTCCAATG |
| AP-1-F | AGAGGCTCATCATCCAGTCTAGC |
| AP-1-R | TGCTGTTGTTGATGGTCGTTT |
| Cycsb-F | ACTGGACAGGCAGAGGGGTAT |
| Cycsb-R | TGGAATGTACTTCTTGGGGTTCT |

**Table S2** Apoptotic and metabolic related GO terms with number of DEGs ≥2

| GO_ID | Description | DEGs | p-value |
| --- | --- | --- | --- |
| GO:0043170 | macromolecule metabolic process | 20 | 0.030657 |
| GO:0034641 | cellular nitrogen compound metabolic process | 16 | 0.011808 |
| GO:1901360 | organic cyclic compound metabolic process | 16 | 0.013107 |
| GO:0006807 | nitrogen compound metabolic process | 16 | 0.030662 |
| GO:0019222 | regulation of metabolic process | 15 | 4.62E-05 |
| GO:0006139 | nucleobase-containing compound metabolic process | 15 | 0.018935 |
| GO:0006725 | cellular aromatic compound metabolic process | 15 | 0.02631 |
| GO:0046483 | heterocycle metabolic process | 15 | 0.027568 |
| GO:0016070 | RNA metabolic process | 14 | 0.001049 |
| GO:0090304 | nucleic acid metabolic process | 14 | 0.01417 |
| GO:0060255 | regulation of macromolecule metabolic process | 13 | 0.000778 |
| GO:0031323 | regulation of cellular metabolic process | 13 | 0.000951 |
| GO:0080090 | regulation of primary metabolic process | 12 | 0.002581 |
| GO:0051252 | regulation of RNA metabolic process | 11 | 0.000629 |
| GO:0019219 | regulation of nucleobase-containing compound metabolic process | 11 | 0.001149 |
| GO:0051171 | regulation of nitrogen compound metabolic process | 11 | 0.001197 |
| GO:0006796 | phosphate-containing compound metabolic process | 8 | 0.005733 |
| GO:0006793 | phosphorus metabolic process | 8 | 0.006736328 |
| GO:0044281 | small molecule metabolic process | 7 | 0.020666516 |
| GO:0005975 | carbohydrate metabolic process | 5 | 0.017011422 |
| GO:0006915 | apoptotic process | 4 | 0.023991557 |
| GO:0031325 | positive regulation of cellular metabolic process | 4 | 0.041633528 |
| GO:0042981 | regulation of apoptotic process | 3 | 0.003916428 |
| GO:0008203 | cholesterol metabolic process | 2 | 0.001815538 |
| GO:0016125 | sterol metabolic process | 2 | 0.003819836 |
| GO:0016071 | mRNA metabolic process | 2 | 0.0110524 |
| GO:0006066 | alcohol metabolic process | 2 | 0.011062979 |
| GO:0008202 | steroid metabolic process | 2 | 0.012370543 |
| GO:1901615 | organic hydroxy compound metabolic process | 2 | 0.016673223 |
| GO:0044262 | cellular carbohydrate metabolic process | 2 | 0.016985981 |
| GO:0006631 | fatty acid metabolic process | 2 | 0.033026187 |
| GO:0051173 | positive regulation of nitrogen compound metabolic process | 2 | 0.048418674 |

**Table S3** Enriched GO terms for the up-regulated DEGs after the Ich infections

| GO_ID | Description | T1 | T2 | T3 |
| --- | --- | --- | --- | --- |
| GO:0043280 | positive regulation of cysteine-type endopeptidase activity involved in apoptotic process | 0.003926 | NA | NA |
| GO:0009257 | 10-formyltetrahydrofolate biosynthetic process | 0.003926 | NA | NA |
| GO:2001056 | positive regulation of cysteine-type endopeptidase activity | 0.003926 | NA | NA |
| GO:0009256 | 10-formyltetrahydrofolate metabolic process | 0.003926 | NA | NA |
| GO:0004477 | methenyltetrahydrofolate cyclohydrolase activity | 0.007837 | NA | NA |
| GO:0071353 | cellular response to interleukin-4 | 0.007837 | NA | NA |
| GO:0010950 | positive regulation of endopeptidase activity | 0.007837 | NA | NA |
| GO:0010952 | positive regulation of peptidase activity | 0.007837 | NA | NA |
| GO:0009396 | folic acid-containing compound biosynthetic process | 0.007837 | NA | NA |
| GO:0046654 | tetrahydrofolate biosynthetic process | 0.007837 | NA | NA |
| GO:0004674 | protein serine/threonine kinase activity | 0.009479 | 8.86E-05 | NA |
| GO:0046653 | tetrahydrofolate metabolic process | 0.011734 | NA | NA |
| GO:0017049 | GTP-Rho binding | 0.015616 | NA | NA |
| GO:0043650 | dicarboxylic acid biosynthetic process | 0.015616 | NA | NA |
| GO:0043281 | regulation of cysteine-type endopeptidase activity involved in apoptotic process | 0.019484 | NA | NA |
| GO:2000116 | regulation of cysteine-type endopeptidase activity | 0.019484 | NA | NA |
| GO:0000978 | RNA polymerase II core promoter proximal region sequence-specific DNA binding | 0.027175 | 0.064898 | NA |
| GO:0000987 | core promoter proximal region sequence-specific DNA binding | 0.027175 | 0.064898 | NA |
| GO:0001159 | core promoter proximal region DNA binding | 0.027175 | 0.064898 | NA |
| GO:0052548 | regulation of endopeptidase activity | 0.027175 | NA | NA |
| GO:0042775 | mitochondrial ATP synthesis coupled electron transport | 0.031 | NA | NA |
| GO:0017048 | Rho GTPase binding | 0.046154 | NA | NA |
| GO:0008203 | cholesterol metabolic process | NA | 0.001816 | NA |
| GO:0006468 | protein phosphorylation | 0.364444 | 0.004331 | NA |
| GO:0016435 | rRNA (guanine) methyltransferase activity | NA | 0.009534 | NA |
| GO:0004613 | phosphoenolpyruvate carboxykinase (GTP) activity | NA | 0.009534 | 0.002243 |
| GO:0000185 | activation of MAPKKK activity | NA | 0.009534 | NA |
| GO:0070328 | triglyceride homeostasis | NA | 0.009534 | NA |
| GO:0051006 | positive regulation of lipoprotein lipase activity | NA | 0.009534 | NA |
| GO:0060379 | cardiac muscle cell myoblast differentiation | NA | 0.009534 | NA |
| GO:2000725 | regulation of cardiac muscle cell differentiation | NA | 0.009534 | NA |
| GO:0055090 | acylglycerol homeostasis | NA | 0.009534 | NA |
| GO:0032374 | regulation of cholesterol transport | NA | 0.009534 | NA |
| GO:0061365 | positive regulation of triglyceride lipase activity | NA | 0.009534 | NA |
| GO:0034434 | sterol esterification | NA | 0.009534 | NA |
| GO:0034435 | cholesterol esterification | NA | 0.009534 | NA |
| GO:0018105 | peptidyl-serine phosphorylation | NA | 0.01823 | NA |
| GO:0018209 | peptidyl-serine modification | NA | 0.01823 | NA |
| GO:1902475 | L-alpha-amino acid transmembrane transport | NA | 0.01898 | NA |
| GO:0070476 | rRNA (guanine-N7)-methylation | NA | 0.01898 | NA |
| GO:0019433 | triglyceride catabolic process | NA | 0.01898 | NA |
| GO:0033344 | cholesterol efflux | NA | 0.01898 | NA |
| GO:0042632 | cholesterol homeostasis | NA | 0.01898 | NA |
| GO:0043691 | reverse cholesterol transport | NA | 0.01898 | NA |
| GO:0070475 | rRNA base methylation | NA | 0.01898 | NA |
| GO:0055092 | sterol homeostasis | NA | 0.01898 | NA |
| GO:0051004 | regulation of lipoprotein lipase activity | NA | 0.01898 | NA |
| GO:0010923 | negative regulation of phosphatase activity | NA | 0.028337 | NA |
| GO:0070700 | BMP receptor binding | NA | 0.028337 | NA |
| GO:0008649 | rRNA methyltransferase activity | NA | 0.028337 | NA |
| GO:0009452 | 7-methylguanosine RNA capping | NA | 0.028337 | NA |
| GO:0036260 | RNA capping | NA | 0.028337 | NA |
| GO:0006641 | triglyceride metabolic process | NA | 0.028337 | NA |
| GO:0046464 | acylglycerol catabolic process | NA | 0.028337 | NA |
| GO:0060193 | positive regulation of lipase activity | NA | 0.028337 | NA |
| GO:0001934 | positive regulation of protein phosphorylation | NA | 0.028769 | NA |
| GO:0009749 | response to glucose | NA | 0.037606 | 0.008946 |
| GO:0000082 | G1/S transition of mitotic cell cycle | NA | 0.037606 | NA |
| GO:0009746 | response to hexose | NA | 0.037606 | 0.008946 |
| GO:0045661 | regulation of myoblast differentiation | NA | 0.037606 | NA |
| GO:0048922 | posterior lateral line neuromast deposition | NA | 0.046789 | NA |
| GO:0010389 | regulation of G2/M transition of mitotic cell cycle | NA | 0.046789 | NA |
| GO:0006695 | cholesterol biosynthetic process | NA | 0.046789 | NA |
| GO:0000154 | rRNA modification | NA | 0.046789 | NA |
| GO:0010830 | regulation of myotube differentiation | NA | 0.046789 | NA |
| GO:1902749 | regulation of cell cycle G2/M phase transition | NA | 0.046789 | NA |
| GO:0006813 | potassium ion transport | NA | NA | 0.000857 |
| GO:0015272 | ATP-activated inward rectifier potassium channel activity | NA | NA | 0.002243 |
| GO:0005249 | voltage-gated potassium channel activity | NA | NA | 0.002487 |
| GO:0005267 | potassium channel activity | NA | NA | 0.00378 |
| GO:0015079 | potassium ion transmembrane transporter activity | NA | NA | 0.004142 |
| GO:0022843 | voltage-gated cation channel activity | NA | NA | 0.005324 |
| GO:0005244 | voltage-gated ion channel activity | NA | NA | 0.009958 |
| GO:0022832 | voltage-gated channel activity | NA | NA | 0.010521 |
| GO:0005242 | inward rectifier potassium channel activity | NA | NA | 0.013391 |
| GO:0005261 | cation channel activity | NA | NA | 0.014194 |
| GO:0004402 | histone acetyltransferase activity | NA | 0.064898 | 0.015607 |
| GO:0006094 | gluconeogenesis | NA | 0.064898 | 0.015607 |
| GO:0019319 | hexose biosynthetic process | NA | 0.064898 | 0.015607 |
| GO:0017017 | MAP kinase tyrosine/serine/threonine phosphatase activity | NA | 0.073825 | 0.017819 |
| GO:0033549 | MAP kinase phosphatase activity | NA | 0.073825 | 0.017819 |
| GO:0015077 | monovalent inorganic cation transmembrane transporter activity | NA | NA | 0.017984 |
| GO:0046873 | metal ion transmembrane transporter activity | NA | NA | 0.029776 |
| GO:0008080 | N-acetyltransferase activity | NA | 0.125661 | 0.030991 |
| GO:0008138 | protein tyrosine/serine/threonine phosphatase activity | NA | 0.142299 | 0.035346 |
| GO:0006006 | glucose metabolic process | NA | 0.150502 | 0.037516 |
| GO:0022890 | inorganic cation transmembrane transporter activity | NA | NA | 0.037806 |

T1: p-value of control vs. early-infected stage, T2: p-value of control vs. late-infected stage, T3: p-value of early-infected stage vs. late-infected stage. NA means no enriched.

**Table S4** Enriched GO terms for the down-regulated DEGs after the Ich infections

| GO_ID | Description | T1 | T2 | T3 |
| --- | --- | --- | --- | --- |
| GO:0061158 | 3'-UTR-mediated mRNA destabilization | 0.009327 | 0.021201 | NA |
| GO:0006777 | Mo-molybdopterin cofactor biosynthetic process | 0.013958 | NA | NA |
| GO:0031034 | myosin filament assembly | 0.013958 | NA | NA |
| GO:0030240 | skeletal muscle thin filament assembly | 0.01857 | NA | NA |
| GO:0008253 | 5'-nucleotidase activity | 0.01857 | 0.04196 | NA |
| GO:0034975 | protein folding in endoplasmic reticulum | 0.01857 | NA | NA |
| GO:0008252 | nucleotidase activity | 0.01857 | 0.04196 | NA |
| GO:0042470 | melanosome | 0.02316 | NA | NA |
| GO:0003730 | mRNA 3'-UTR binding | 0.02316 | 0.052177 | NA |
| GO:0004993 | serotonin receptor activity | 0.045805 | 0.101676 | NA |
| GO:0042593 | glucose homeostasis | NA | 0.002264 | NA |
| GO:0016607 | nuclear speck | NA | 0.009352 | 0.025889 |
| GO:0004320 | oleoyl-[acyl-carrier-protein] hydrolase activity | NA | 0.010656 | NA |
| GO:0004317 | 3-hydroxypalmitoyl-[acyl-carrier-protein] dehydratase activity | NA | 0.010656 | NA |
| GO:0004313 | [acyl-carrier-protein] S-acetyltransferase activity | NA | 0.010656 | NA |
| GO:0016296 | palmitoyl-[acyl-carrier-protein] hydrolase activity | NA | 0.010656 | NA |
| GO:0004314 | [acyl-carrier-protein] S-malonyltransferase activity | NA | 0.010656 | NA |
| GO:0016295 | myristoyl-[acyl-carrier-protein] hydrolase activity | NA | 0.010656 | NA |
| GO:0004315 | 3-oxoacyl-[acyl-carrier-protein] synthase activity | NA | 0.010656 | NA |
| GO:0016418 | S-acetyltransferase activity | NA | 0.010656 | NA |
| GO:0016419 | S-malonyltransferase activity | NA | 0.010656 | NA |
| GO:0003729 | mRNA binding | 0.067945 | 0.010718 | NA |
| GO:0005506 | iron ion binding | NA | 0.020355 | 0.004431 |
| GO:0015199 | amino-acid betaine transmembrane transporter activity | NA | 0.021201 | NA |
| GO:0006517 | protein deglycosylation | NA | 0.021201 | NA |
| GO:0008455 | alpha-1,6-mannosylglycoprotein 2-beta-N-acetylglucosaminyltransferase activity | NA | 0.021201 | NA |
| GO:0000381 | regulation of alternative mRNA splicing, via spliceosome | NA | 0.021201 | 0.003736 |
| GO:0010133 | proline catabolic process to glutamate | NA | 0.021201 | NA |
| GO:0000380 | alternative mRNA splicing, via spliceosome | NA | 0.021201 | 0.003736 |
| GO:0006067 | ethanol metabolic process | NA | 0.021201 | NA |
| GO:0046085 | adenosine metabolic process | NA | 0.031635 | NA |
| GO:0015651 | quaternary ammonium group transmembrane transporter activity | NA | 0.031635 | NA |
| GO:0072341 | modified amino acid binding | NA | 0.031635 | NA |
| GO:0048024 | regulation of mRNA splicing, via spliceosome | NA | 0.031635 | 0.005599 |
| GO:0016556 | mRNA modification | NA | 0.031635 | 0.005599 |
| GO:0009065 | glutamine family amino acid catabolic process | NA | 0.031635 | NA |
| GO:0030073 | insulin secretion | NA | 0.04196 | NA |
| GO:0006635 | fatty acid beta-oxidation | NA | 0.04196 | NA |
| GO:0046548 | retinal rod cell development | NA | 0.04196 | NA |
| GO:0072349 | modified amino acid transmembrane transporter activity | NA | 0.04196 | NA |
| GO:0006397 | mRNA processing | NA | 0.170804 | 0.007099 |

T1: p-value of control vs. early-infected stage, T2: p-value of control vs. late-infected stage, T3: p-value of early-infected stage vs. late-infected stage. NA means no enriched.

**Table S5** Enriched KEGG pathways for the up-regulated DEGs after the Ich infections

| Pathway ID | Description | T1 | T2 | T3 |
| --- | --- | --- | --- | --- |
| ko05168 | Herpes simplex infection | 0.000104 | 0.172203 | 0.187427 |
| ko04975 | Fat digestion and absorption | 0.000264 | 0.000396 | NA |
| ko04214 | Apoptosis - fly | 0.008599 | NA | NA |
| ko04977 | Vitamin digestion and absorption | 0.008599 | 0.010156 | NA |
| ko04215 | Apoptosis - multiple species | 0.010856 | NA | NA |
| ko00660 | C5-Branched dibasic acid metabolism | 0.010871 | NA | NA |
| ko04380 | Osteoclast differentiation | 0.011223 | 0.008217 | NA |
| ko04668 | TNF signaling pathway | 0.011954 | 0.261834 | NA |
| ko05134 | Legionellosis | 0.016472 | 0.291338 | 0.204128 |
| ko00601 | Glycosphingolipid biosynthesis - lacto and neolacto series | 0.020062 | 0.356532 | NA |
| ko04657 | IL-17 signaling pathway | 0.022839 | 0.001403 | 0.227937 |
| ko04210 | Apoptosis | 0.03058 | 0.687265 | NA |
| ko05164 | Influenza A | 0.032459 | 0.345797 | 0.080689 |
| ko05161 | Hepatitis B | 0.03427 | 0.037152 | 0.407582 |
| ko05132 | Salmonella infection | 0.034893 | 0.056079 | NA |
| ko00920 | Sulfur metabolism | 0.042786 | NA | NA |
| ko04621 | NOD-like receptor signaling pathway | 0.046398 | 0.016379 | 0.304139 |
| ko03018 | RNA degradation | 0.050439 | 0.026102 | 0.298554 |
| ko04115 | p53 signaling pathway | 0.081701 | 0.015358 | 0.017402 |
| ko00830 | Retinol metabolism | 0.333131 | 0.04764 | 0.158272 |
| ko04068 | FoxO signaling pathway | NA | 0.003317 | 0.007902 |
| ko04922 | Glucagon signaling pathway | NA | 0.024213 | 0.004992 |
| ko05219 | Bladder cancer | NA | 0.029292 | 0.132364 |
| ko00010 | Glycolysis / Gluconeogenesis | NA | 0.234555 | 0.016287 |
| ko04920 | Adipocytokine signaling pathway | NA | 0.101558 | 0.023442 |
| ko04350 | TGF-beta signaling pathway | NA | 0.176377 | 0.037666 |
| ko04213 | Longevity regulating pathway - multiple species | NA | 0.590868 | 0.015204 |
| ko00750 | Vitamin B6 metabolism | NA | NA | 0.016139 |

T1: p-value of control vs. early-infected stage, T2: p-value of control vs. late-infected stage, T3: p-value of early-infected stage vs. late-infected stage. NA means no enriched.

**Table S6** Enriched KEGG pathways for the down-regulated DEGs after the Ich infections

| Pathway ID | Description | T1 | T2 | T3 |
| --- | --- | --- | --- | --- |
| ko04141 | Protein processing in endoplasmic reticulum | 2.09E-06 | 0.067602 | 0.409837 |
| ko04610 | Complement and coagulation cascades | 8.75E-05 | 0.000224 | 0.054116 |
| ko04612 | Antigen processing and presentation | 0.000625 | 0.016682 | 0.312771 |
| ko00330 | Arginine and proline metabolism | 0.000872 | 0.202295 | NA |
| ko05215 | Prostate cancer | 0.004534 | 0.042209 | NA |
| ko00790 | Folate biosynthesis | 0.005535 | 0.198243 | NA |
| ko04659 | Th17 cell differentiation | 0.006989 | 0.20616 | NA |
| ko01100 | Metabolic pathways | 0.010729 | 0.006866 | 0.346098 |
| ko04213 | Longevity regulating pathway - multiple species | 0.012256 | 0.014456 | NA |
| ko05145 | Toxoplasmosis | 0.020911 | 0.335737 | NA |
| ko04915 | Estrogen signaling pathway | 0.024968 | 0.171304 | NA |
| ko00040 | Pentose and glucuronate interconversions | 0.026664 | 0.013382 | 0.087285 |
| ko04151 | PI3K-Akt signaling pathway | 0.027511 | 0.555691 | NA |
| ko04657 | IL-17 signaling pathway | 0.028076 | 0.367909 | NA |
| ko04658 | Th1 and Th2 cell differentiation | 0.02947 | 0.194311 | NA |
| ko04914 | Progesterone-mediated oocyte maturation | 0.030732 | 0.384406 | NA |
| ko00053 | Ascorbate and aldarate metabolism | 0.03268 | 0.017984 | 0.096939 |
| ko05323 | Rheumatoid arthritis | 0.035469 | 0.435465 | NA |
| ko00350 | Tyrosine metabolism | 0.04469 | 0.140729 | NA |
| ko00860 | Porphyrin and chlorophyll metabolism | 0.046111 | 0.004525 | 0.115948 |
| ko00982 | Drug metabolism - cytochrome P450 | 0.049003 | 6.63E-05 | 0.007181 |
| ko04975 | Fat digestion and absorption | NA | 0.028221 | 0.114064 |
| ko00061 | Fatty acid biosynthesis | 0.142784 | 1.06E-05 | 0.053704 |
| ko00980 | Metabolism of xenobiotics by cytochrome P450 | 0.053462 | 8.75E-05 | 0.125305 |
| ko05204 | Chemical carcinogenesis | 0.070943 | 0.000215 | 0.145552 |
| ko01212 | Fatty acid metabolism | 0.366731 | 0.000267 | 0.150994 |
| ko04940 | Type I diabetes mellitus | 0.066102 | 0.002367 | 0.043682 |
| ko00071 | Fatty acid degradation | 0.286821 | 0.004251 | 0.114064 |
| ko05332 | Graft-versus-host disease | 0.187553 | 0.004696 | 0.032766 |
| ko00983 | Drug metabolism - other enzymes | 0.056511 | 0.006783 | 0.000353 |
| ko00140 | Steroid hormone biosynthesis | 0.061196 | 0.007943 | 0.134566 |
| ko00830 | Retinol metabolism | 0.070943 | 0.010639 | 0.145552 |
| ko03320 | PPAR signaling pathway | 0.526934 | 0.015599 | 0.029265 |
| ko05322 | Systemic lupus erythematosus | 0.083509 | 0.01615 | 0.11795 |
| ko05330 | Allograft rejection | 0.280925 | 0.017106 | 0.054116 |
| ko04152 | AMPK signaling pathway | 0.283111 | 0.017538 | NA |
| ko05320 | Autoimmune thyroid disease | 0.309286 | 0.023313 | 0.061325 |
| ko04974 | Protein digestion and absorption | 0.116236 | 0.029623 | 0.067669 |
| ko04972 | Pancreatic secretion | 0.337428 | 0.030879 | 0.068845 |
| ko03018 | RNA degradation | NA | 0.830278 | 0.041206 |
| ko04744 | Phototransduction | NA | 0.148696 | 0.006951 |
| ko05020 | Prion diseases | 0.06763986 | 0.5676033 | 0.01020537 |
| ko00232 | Caffeine metabolism | NA | NA | 0.0209987 |
| ko05166 | HTLV-I infection | 0.6116947 | 0.2742467 | 0.02571632 |
| ko04611 | Platelet activation | NA | 0.685334 | 0.02642322 |
| ko00230 | Purine metabolism | 0.3269641 | 0.3593845 | 0.0353881 |
| ko00310 | Lysine degradation | NA | 0.5506178 | 0.04468717 |
| ko04514 | Cell adhesion molecules (CAMs) | 0.8948862 | 0.7516891 | 0.0456409 |

T1: p-value of control vs. early-infected stage, T2: p-value of control vs. late-infected stage, T3: p-value of early-infected stage vs. late-infected stage. NA means no enriched.

**Table S7** Summary of categorization about identified putative AMPs/AMP precursors

| Types | Number |
| --- | --- |
| Histone | 105 |
| Scolopendin | 15 |
| Hemoglobin | 13 |
| Neuropeptide | 8 |
| Chemokine | 5 |
| Thrombin | 4 |
| Ubiquitin | 4 |
| Beta2-Microglobulin | 2 |
| Defensin | 2 |
| Lysozyme | 2 |
| Skin-PYY | 2 |
| Synuclein | 2 |
| TroTbeta4_fish | 2 |
| Ubiquicidin | 2 |
| Amylin | 1 |
| Amyloid | 1 |
| Antiproteinase | 1 |
| BPTI | 1 |
| CcAMP1_insect | 1 |
| Cerotoxin | 1 |
| GAPDH | 1 |
| Peptide_3910_pig | 1 |
| Phospholipase_A2 | 1 |
| Thymosin | 1 |
